# Consumption of Substances of Abuse during Pregnancy Increases Consumption in Offspring: Possible Underlying Mechanisms

**DOI:** 10.3389/fnut.2016.00011

**Published:** 2016-04-20

**Authors:** Kinning Poon, Sarah F. Leibowitz

**Affiliations:** ^1^Laboratory of Behavioral Neurobiology, The Rockefeller University, New York, NY, USA

**Keywords:** prenatal fat, prenatal ethanol, prenatal nicotine, inflammation, ingestive behavior

## Abstract

Correlative human observational studies on substances of abuse have been highly dependent on the use of rodent models to determine the neuronal and molecular mechanisms that control behavioral outcomes. This is particularly true for gestational exposure to non-illicit substances of abuse, such as excessive dietary fat, ethanol, and nicotine, which are commonly consumed in our society. Exposure to these substances during the prenatal period has been shown in offspring to increase their intake of these substances, induce other behavioral changes, and affect neurochemical systems in several brain areas that are known to control behavior. More importantly, emerging studies are linking the function of the immune system to these neurochemicals and ingestion of these abused substances. This review article will summarize the prenatal rodent models used to study developmental changes in offspring caused by prenatal exposure to dietary fat, ethanol, or nicotine. We will discuss the various techniques used for the administration of these substances into rodents and summarize the published outcomes induced by prenatal exposure to these substances. Finally, this review will cover some of the recent evidence for the role of immune factors in causing these behavioral and neuronal changes.

## Introduction

Scientific research has relied heavily on the use of animal models to identify various characteristics of diseases and disorders found in humans. These animal models serve an important purpose when there is limited ability to ethically evaluate such disorders in humans. Most limiting in human research are studies of embryonic development and the effects produced by exposure to substances of abuse, such as alcohol, nicotine, and dietary fat, which occur as a result of voluntary maternal consumption. In humans, ingestion of alcohol during pregnancy triggers neurological disorders and increases the risk of fetal alcohol syndrome in the offspring (1, 2), effects subsequently confirmed and characterized using animal models (3–5). Also, smoking during pregnancy increases the risk of a decrease in birth weight (6, 7) and multiple behavioral problems (8), including attention deficit disorders (9) and increased propensity to abuse drugs (10, 11). In human observational studies, increased intake of dietary fats and obesity during pregnancy are found to increase the risk for dietary obesity in offspring (12–14).

Further testing of these physiological and behavioral changes using animal models exposed to substances of abuse have revealed disturbances in the development of neuronal circuits that modulate both homeostatic and reward pathways (15–17). The main players involved include a variety of neuropeptides that are found in various regions of the hypothalamus and forebrain and are shown to modulate neuronal function that may ultimately contribute to the behavioral changes in offspring. These behavioral changes include an increased propensity to ingest these substances of abuse (15–18), with a significant crossover effect from one substance to another (19). Although great strides have been taken to characterize these changes in neurochemical systems that control behavior, the molecular mechanisms involved in producing these disturbances in the brain have yet to be determined.

In addition to these neurochemicals, the field of ingestive behavior has recently focused attention on immunology. These new studies build on prior research of neurological disorders and neurodegenerative diseases, which show the immune system to play a large part in the health, function, and development of neurons and other cell types in the central nervous system (20–24), along with the development of embryos (25, 26). Recently, both human and animal observational studies have demonstrated that exposure to these substances of abuse, in addition to altering classical neuropeptide and neurotransmitter systems, also disturbs inflammatory systems in key regions of the brain that control ingestion and related behaviors (17, 27, 28). Prenatal inflammation itself has been shown to increase the risk of developing neurological disorders and diseases, such as autism (29, 30) and other psychiatric disorders (31), which the offspring are at a higher risk of developing when exposed during gestation to substances of abuse (32–35).

To understand these neurochemical and immune systems affected by prenatal substance exposure and their possible role in promoting consummatory and other behaviors in the offspring, the use of animal models involving prenatal manipulations is clearly essential. This review will cover the current techniques used to perform prenatal studies using rodent models and their general conclusions about the neuronal changes induced by embryonic exposure to environmental substances. It will also summarize the current research linking these neuronal and neurochemical changes to inflammatory systems, focusing on the three most commonly abused substances, dietary fat, alcohol, and nicotine.

## Experimental Methods Used to Introduce Environmental Factors into Pregnant Rats

There are a few factors that must be taken into consideration when designing an experiment using a rodent model. The first is to choose the appropriate rodent strain to use in your model, with various strains having different preferences for different substances. Rat strains with a preference for dietary fats include Sprague-Dawley (36), Brattleboro (37, 38), and Zucker (39), with the latter having an obese phenotype. In alcohol studies, several different strains are used, including outbred rats such as Wistar (40) and Long-Evans (41), and also genetically modified rats that have increased alcohol intake, such as ALKO alcohol (42), high alcohol drinking or HAD (40), and Sardinian preferring (sP) (43) rats. In nicotine research, rats have been found to show differences in behavioral effects between different strains (44, 45) that are attributed to genetic variability. Some of the rat strains used in nicotine studies include Sprague-Dawley (46), Long-Evans (47), Lewis (48), Holtzman (49, 50), and Wistar (51).

The second factor is choosing the method for administering the substance of abuse. This can be broken down into two main paradigms, forced or choice. The forced paradigm does not give the animal a choice in intake, with the substance being the only option or its administration being forced. This is in contrast to a choice paradigm, whereby the animal has one or more competing substances to choose from, with one of the options generally being a control substance, such as chow or water. Studies on dietary fat have used both choice and forced paradigms, with some reports using a combination of the two. Generally, a high-fat and a low-fat diet are made available to the rat, with intake measured daily (52, 53). In combination paradigms, rats are exposed to the high-fat diet in conjunction with their usual diet for a period of several days until acclimation to the new diet is achieved, after which the high-fat diet is given as the only choice (52, 53). Under forced conditions, rats may be given an oil emulsion *via* oral gavage (54). Studies of ethanol and nicotine, in choice paradigms have used both methods of self-administration (55, 56) and two bottle conditions (57–59). Generally, the concentration of ethanol or nicotine is given in intervals, ranging from a low to high concentration, until the desired concentration is reached (60, 61), with some groups combining palatable sucrose with ethanol or nicotine until voluntary drinking of the drug is established (62). Forced exposure methods, in contrast, include oral gavage, direct injection into the peritoneal cavity (61, 63), intravenous infusion (64), a liquid diet (65), or having the substance as a sole liquid source (66–69).

In studies relating inflammation to ingestive behavior, a specific inflammatory mediator or an agent that induces inflammation, such as lipopolysaccharide, can be administered to any area of the rat through injection. This includes systemic infusion (70), intraperitoneal injection (71, 72), or use of an osmotic mini-pump (73–75).

**Figure 1:**
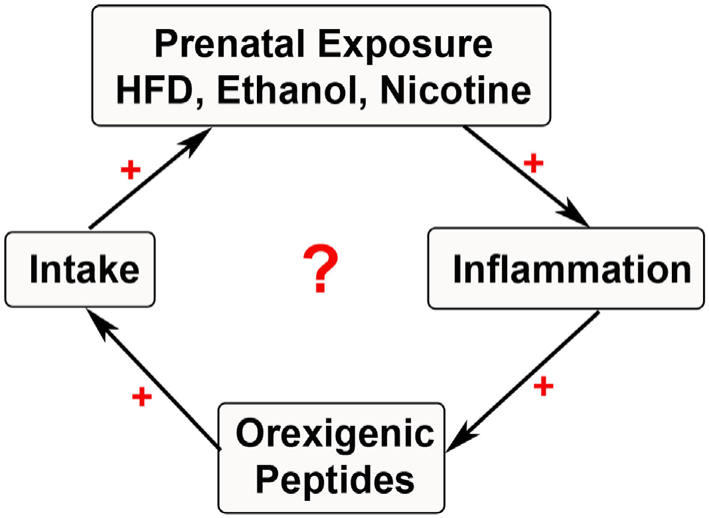
**Cycle of substance exposure**. The schematic depicts the current hypothesis of a simplified positive feedback loop involving prenatal exposure to substances of abuse that stimulate inflammatory systems. This inflammation may be involved in stimulating neuropeptides that further increase ingestive behavior, thus leading to a cyclical increase in exposure during the prenatal period with negative outcomes in the offspring.

These methods are only a brief summary, with a wide range of models and rodents used to study the effects of prenatal exposure. Once a particular model is well established, further measurements of behavior in tissues and cells of different type can be extensively performed. The sections below will focus on our current understanding of how prenatal exposure to substances of abuse affect neuronal systems that control behavior in offspring and how the inflammatory response may be a factor in promoting those changes (Figure 1).

## Prenatal HFD Exposure

**Figure 2:**
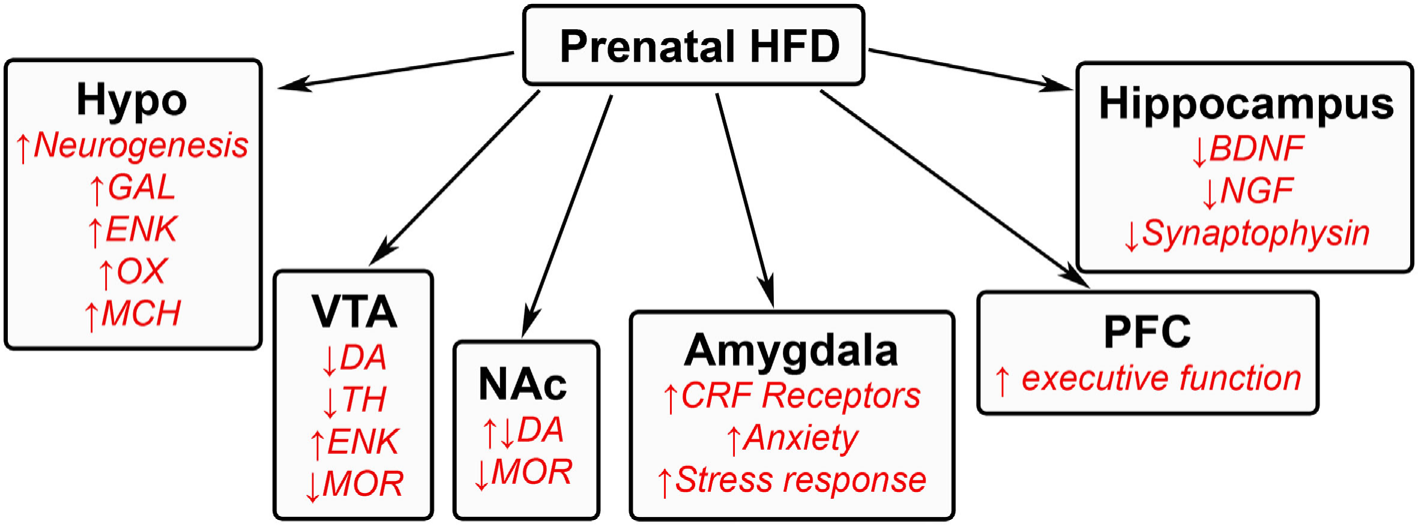
**Effects of prenatal HFD exposure on offspring brain**. A schematic summarizing some of the changes that occur in the brains of offspring after being exposed to a HFD during gestation. GAL, galanin; ENK, enkephalin; OX, orexin; MCH, melanin-concentrating hormone; DA, dopamine; TH, tyrosine hydroxylase; MOR, μ-opioid receptor; CRF, corticotrophin releasing factor; BDNF, brain-derived neurotrophic factor; NGF, nerve growth factor.

Animal models investigating the effects of excessive HFD intake during pregnancy have revealed several changes in both the physiology and behavior of offspring. Prenatal exposure to a HFD has been shown to induce several effects in offspring. These include increased body weight, faster weight gain, and larger fat pads (15, 76–78), as well as behavioral changes that include increased ingestion (15, 52, 76), autism spectrum disorders (32, 33), depression (79), and attention hyperactive disorders (80) along with a decrease in spatial memory acquisition (78, 81). Increased understanding of the neuronal systems involved in invoking these behavioral changes is made possible by the numerous animal models used to study these phenomena. These behavioral changes have been attributed to changes in the neurochemistry of various brain regions involved in homeostatic, reward, emotional, and memory processes (Figure 2) and, more recently, to changes in inflammatory processes.

### Prenatal HFD Exposure Alters Hypothalamic Neurocircuitry

Changes in specific brain areas caused by prenatal HFD exposure seem to control different aspects of HFD intake. The change in homeostatic processes occurs in the hypothalamus, a region important in controlling ingestive behavior. Several lines of evidence show prenatal exposure to a HFD to produce changes in both the developing embryo and in adolescent and adult offspring. These include an increase in the neurogenesis of hypothalamic orexigenic peptide neurons (15, 82), with increased synthesis of the peptides that further induce HFD intake (15). These neuropeptides include galanin and enkephalin in the medial paraventricular nucleus (15, 83), orexin and melanin-concentrating hormone in the perifornical lateral hypothalamus (15), and also ghrelin in the midbrain (84).

### Prenatal HFD Exposure Alters VTA–NA System in Offspring

The centers controlling the rewarding aspects of intake consist of the ventral tegmental area (VTA) and the nucleus accumbens (NA) core and shell, which contain the dopaminergic signaling system, μ-opioid receptors, and glutamatergic inputs that are activated by rewarding substances (85, 86). Similar to drug addiction (85), prolonged intake of a HFD has been shown to block dopamine reuptake and enhance dopaminergic function (87). Similarly, exposure to this diet during the prenatal period has been found in adult offspring to increase the levels and expression of dopamine in the NA core and decrease the expression of tyrosine hydroxylase in the VTA, thus decreasing the formation of dopamine (88, 89). Reduced expression of the μ-opioid receptor (89) and increased levels of enkephalin are also found in the VTA and NA regions, with injection of an enkephalin analog into the NA shown to increase HFD intake (90, 91). Similar changes in dopamine, dopamine transporter, and μ-opioid receptor have been found in other studies using maternal junk food or obesity-prone offspring (92, 93), in addition to a reduction in dopamine release in the nucleus accumbens and other terminal sites of dopamine release (92). These studies suggest that prenatal HFD exposure markedly alters the reward pathway, inducing a compensatory mechanism that leads the offspring to ingest excessive amounts of dietary fat to obtain a rewarding feeling caused by the reduced dopamine function (88, 92). Epigenetic changes involving hypomethylation are also found for the dopamine transporter, μ-opioid receptor, and enkephalin, suggesting long-term changes and consequences in offspring (89). While studies in the VTA–NA system have mostly focused on dopamine and agonists of the μ-opioid receptor, other targets are also involved. These include ghrelin, a neuropeptide, known to stimulate the rewarding effects of food intake (94) and promote the rewarding feeling of food intake (95), which is also abundantly expressed in the VTA and found to increase HFD feeding after injection into the VTA (84).

### Prenatal HFD Has Global Effects on Other Areas of the Brain in Offspring

Other brain regions also show permanent changes that affect behavior. In the hippocampus, prenatal HFD exposure in offspring decreases expression and levels of proteins that are involved in memory function, such as brain-derived neurotrophic factor, nerve growth factor, and synaptophysin, suggesting a delay in memory acquisition (78, 81). The transcription of genes controlling executive function in the prefrontal cortex is also markedly increased by dietary fat in offspring (96). The emotional aspect of feeding, controlled by the amygdala, has been found in adult rats to evoke several changes in neurochemical pathways (97, 98) that in turn may induce changes in anxiety as well as feeding. Although there are only a few studies of prenatal HFD exposure that have examined the amygdala, there is some evidence that altered functioning of this brain region is involved in emotional changes in offspring that may further promote consumption. Exposure to a fat-rich diet during the prenatal period causes in offspring an increase in corticosteroid receptors in the amygdala (99). This exposure also increases anxiety in an open field, an elevated plus maze, and during light–dark transition tasks, while increasing corticosteroid levels in response to stress (99), suggesting an overall increase in the stress response and thus anxiety. These responses have been reported to increase ingestive behavior in attempt to reduce stress (100–102). These global brain changes affecting decision-making may be involved in both the impulsive and rational choice to overeat.

### Prenatal HFD Induces Epigenetic Changes in Offspring

The effect of a HFD during the prenatal period on gene expression in developing neurons is thought to be attributed to epigenetic changes. In human adults, several studies in peripheral tissue reveal alterations in histone modification at promoters of proteins that are affected by dietary fat (103) and in methylation in specific tissue such as skeletal muscle (104). Prenatal exposure to a HFD has also been shown to alter methylation or microRNA expression in placental tissue (105) and adipose tissue (106, 107). That epigenetic changes may be transmitted to offspring is indicated by studies showing a generational effect on specific genes during dietary protein restriction (108, 109). While there is little evidence on the epigenetic effects of prenatal HFD exposure in neurons of embryos and postnatal offspring, several reviews exist that describe global metabolic epigenetic changes in the periphery (110, 111), indicating the need for more such studies in the brain.

### Relationship between Dietary Fat and Inflammation

While several studies examining the effects of acute and chronic inflammatory mediators in adult obese animals have revealed an increase in fat intake and weight gain (112), evidence from prenatal inflammatory studies is more limited. Early findings show chronic HFD intake to induce a systemic low-grade inflammation characterized by an increase in cytokines and chemokines (113, 114). This HFD intake also increases the activation of several inflammatory signaling pathways, such as jun amino-terminal kinases, nuclear factor kappa light-chain enhancer, inhibitor of nuclear factor kappa-B kinase subunit beta, peroxisome proliferator-activator receptor, and toll-like receptors (115–118). Chronic treatment with an agent, such as lipopolysaccharide, that induces inflammation can increase body fat mass and caloric intake, and these effects are exacerbated by a HFD (119), suggesting a strong link between HFD and inflammation. More recent studies have uncovered a major role for chemokines, specifically CCL2, which is affected by a HFD and may also mediate neuronal function. This chemokine has been found early on to be increased in obese animals and during HFD intake (120) and, along with its receptor CCR2, is found in all of the key brain areas involved in HFD ingestion (121, 122). Furthermore, blocking the CCR2 receptor with an antagonist is shown to improve symptoms of obesity and decrease food intake (123, 124). In limited studies, prenatal HFD exposure has been found to increase CCL2 in peripheral organs, such as the liver, in offspring (125).

**Figure 3:**
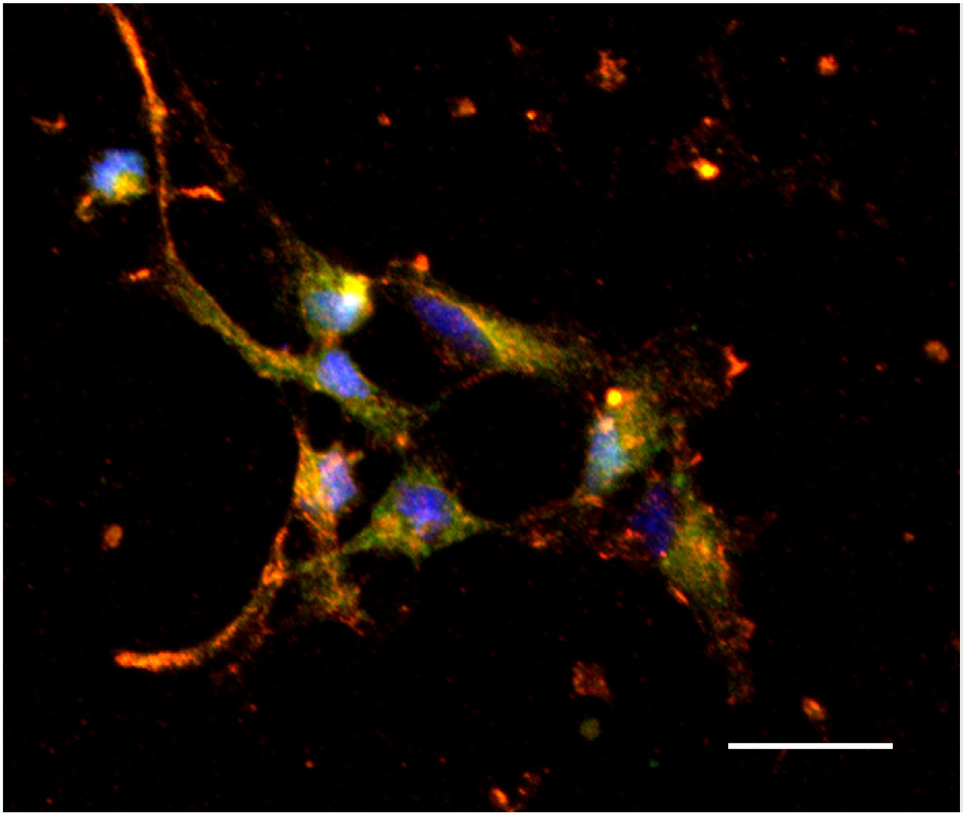
**Colocalization of CCR2 and enkephalin in hypothalamic neurons**. Hypothalamic neurons extracted from chow-exposed embryos showing CCR2 to colocalize with the orexigenic peptide, enkephalin (orange). Scale: 25 μm. Green: enkephalin, red: CCR2, and blue: dapi.

Recent studies from our lab have found a positive relationship between CCL2 and both the migration and expression of orexigenic peptide neurons in primary hypothalamic neurons (126). Exposing cultured embryonic hypothalamic neurons to increasing levels of CCL2 revealed a dose-dependent increase in migration as well as expression of the orexigenic peptides, enkephalin, and galanin in neurons (126). These hypothalamic enkephalin-expressing neurons are found to co-express the receptor, CCR2 (Figure 3), with CCL2 treatment increasing the number of colocalized neurons (126), suggesting an important role for this chemokine in neuronal growth during the prenatal period. In addition, in rats exposed to a HFD during gestation, this chemokine system is found to be greatly altered (127). Prenatal HFD exposure decreases expression of CCL2 while increasing the expression of its receptors, CCR2 and CCR4, in the hypothalamus, and these HFD-exposed neurons are found to exhibit markedly reduced sensitivity to the actions of CCL2 on neuronal migration and peptide expression. With this limited evidence raising new but interesting questions, future studies using the prenatal HFD and prenatal inflammation models should shed light on the molecular mechanisms leading to the neuronal changes and, in turn, altering ingestive behavior in the offspring.

## Prenatal Ethanol Exposure

**Figure 4:**
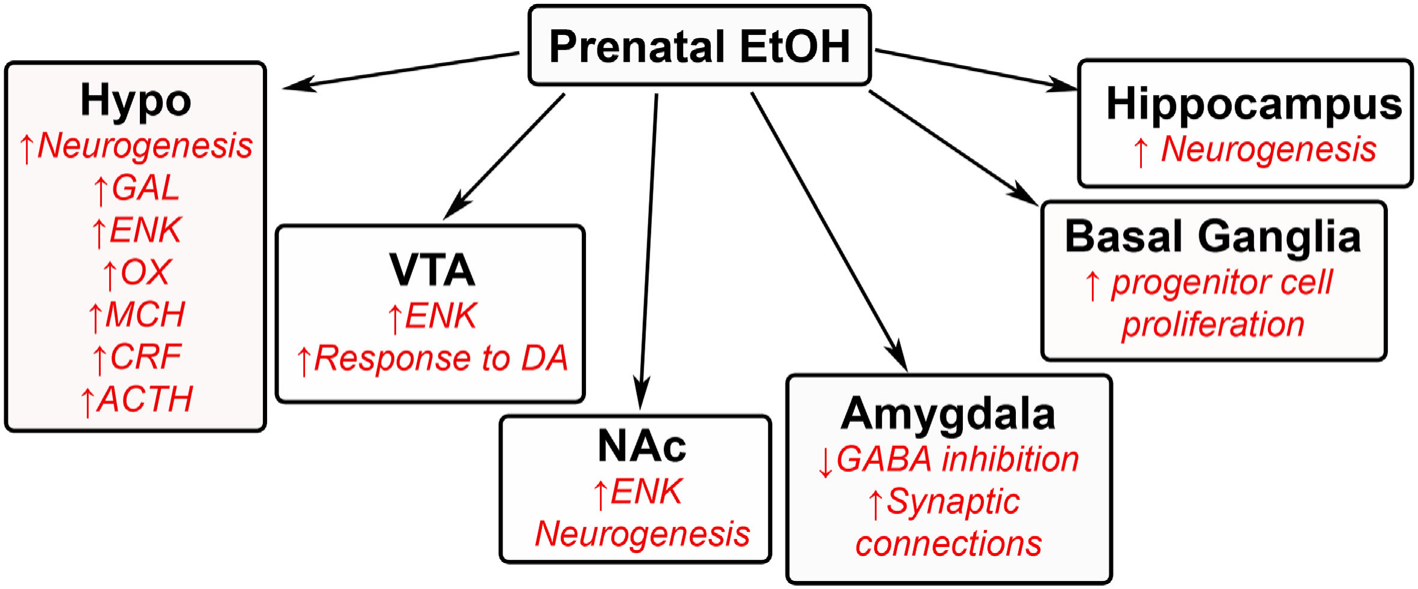
**Effects of prenatal ethanol exposure on offspring brain**. A schematic summarizing some of the changes that occur in the brains of offspring after being exposed to low levels of ethanol during gestation. GAL, galanin; ENK, enkephalin; OX, orexin; MCH, melanin-concentrating hormone; CRF, corticotrophin releasing factor; ACTH, adrenocorticotropic hormone; DA, dopamine; GABA, γ-aminobutyric acid.

Original ethanol studies have shown that exposure during the prenatal period to high levels of alcohol is associated with developing fetal alcohol spectrum disorder in offspring (1–5), with many negative developmental, behavioral, and physiological outcomes (128, 129). These high levels of alcohol, within 20–30% or 6 g/kg/day range, decrease the development of neurons in several brain areas (40, 130–132) and additionally induce epigenetic changes in fetal DNA (133–135). Currently unknown are the effects of low levels of alcohol consumption, within 5% or 1–2 g/kg/day range (16, 136–138), on fetal development and ultimately on offspring behavior. Recent studies have demonstrated that low levels of ethanol exposure during gestation induce several behavioral, neurochemical, and developmental effects, similar to prenatal HFD exposure that are caused by changes in brain regions involved in homeostasis, reward, emotional, memory, and inflammatory processes. These changes are thought to induce excessive drinking (16, 137–139), increased preference (17, 139, 140), and reinforcement (141, 142) for ethanol in offspring during the adolescent period to adulthood. These low levels have also been linked to other behavioral changes, such as hyperactivity (74). While ethanol has several targets in many brain regions, studies of low ethanol levels are lacking. This review will summarize some of the current findings in the field (Figure 4).

### Low Levels of Ethanol Exposure Alters Hypothalamic Neurocircuitry in Offspring

In the hypothalamus, the same orexigenic neuropeptides known to stimulate HFD intake, namely enkephalin, galanin, orexin, and melanin-concentrating hormone, are also found to stimulate ethanol intake [for review, see Ref. (143); (17)]. While different neurochemical systems in the brain are known to be altered by prenatal exposure to ethanol (144–146), the stimulatory effects of prenatal ethanol on these specific neuropeptides are particularly notable, given the potency of their effects on behavior and the sensitivity of the peptide neurons to low doses of ethanol (16, 147). A study from our group has also found low levels of ethanol to increase the genesis of hypothalamic neurons containing enkephalin, orexin, galanin, and melanin-concentrating hormone (16, 17). Additionally, prenatal ethanol exposure is shown to affect stress hormones in the hypothalamus, causing in adolescent and adult offspring an increase in the expression of corticotropin-releasing factor (CRF) in the hypothalamic paraventricular nucleus (144, 148–149) along with levels of corticosterone (144) and also adrenocorticotropic hormone in this same region (144, 150). Prenatal ethanol also increases the levels of these peptides and hormones in response to stress (151–154), with increased stress linked to further consummatory behavior (155–157). Not surprisingly, the CRF system has been linked to addiction of other substances of abuse (158), including dietary fat.

### Low Levels of Ethanol Exposure Alters VTA–NA Center in Offspring

Several studies have linked low levels of ethanol during the prenatal period to changes in the mesolimbic area. Increased neurogenesis of enkephalin neurons is found in the NA shell (16), with overall increased levels of enkephalin in both the VTA (159) and NA core (147, 160). These changes may significantly increase ethanol intake in offspring, as high levels of enkephalin have been shown to activate dopamine terminals in the NA (161, 162). The effects of prenatal ethanol on the dopaminergic system in these brain regions are also significant, with the VTA having an increased response to dopaminergic agonists and the NA having increased sensitivity to the stimulatory effects of alcohol in offspring (137, 163, 164). Although ghrelin has been found to be involved in the rewarding feeling of alcohol (165), there are currently no studies on how low ethanol levels during the prenatal period affects this peptide and other neurochemical systems in these brain regions of offspring.

### Prenatal Ethanol Has Global Effects on Other Areas of the Brain in Offspring

While there exists plenty of research describing the effects of high gestational ethanol exposure on the developing brain, there are only a few studies measuring the effects of low ethanol exposure on other brain areas not discussed above. Some of the findings include an ethanol-induced increase in progenitor cell proliferation in the basal ganglia (166) and a decrease in neural activity in the infralimbic cortex (164). They also include increased neurogenesis in regions of the hippocampus (167). The amygdala has been suggested to be affected by low levels of ethanol during the prenatal period. Offspring exposed to low ethanol display anxiety-like behavior when exposed to stressful conditions, and this behavior has been related to both an increase in synaptic connectivity in the basolateral amygdala (168) and a decrease in GABA inhibition (169), both of which stimulate the excitability of the amygdala (168, 169). Further studies on the effects of low ethanol concentrations in these other brain regions are needed to determine the extent of ethanol’s action on neuronal development throughout the brain.

### Prenatal Ethanol Induces Epigenetic Changes in Offspring

There are several studies that reveal high ethanol exposure during the prenatal period to induce dramatic epigenetic changes in offspring. The adult liver provides a clear example, with high levels of ethanol exposure found to alter DNA methylation related to alcoholic liver disease (170–172). Also, chronic maternal ethanol exposure is shown to decrease methylation at a gene called agouti viable yellow, which affects the color of their coat, that is passed down to offspring (173), while acute prenatal exposure to high levels of ethanol globally causes hypomethylation of DNA in embryos (133). Long-term prenatal exposure to high ethanol levels also induces changes in methylation and microRNA in hippocampal neurons (174). In light of these studies of high ethanol exposure, further investigations of epigenetic effects are clearly needed involving low concentrations of ethanol, which as described above have strong, stimulatory effects on neuronal development in the brain.

### Relationship between Ethanol and Inflammation

Although only a few studies exist, ethanol intake has also been linked to inflammatory systems. The most commonly studied peripheral organ is the liver, with excessive drinking linked to alcoholic liver disease that increases inflammatory mediators (175, 176). More recent studies in adult animals have also shown ethanol exposure to stimulate inflammatory systems in the central nervous system. Endotoxin treatment after ethanol exposure has been found to induce a long-term inflammatory state in the brain (177) and increase nitric oxide synthase and cyclooxygenase, which lead to inflammation (178). This increase in inflammation has also been detected in offspring after prenatal exposure. Similar to prenatal HFD exposure, our lab recently found prenatal ethanol to induce several changes in the CCL2 chemokine system. We found low levels of ethanol during gestation to increase in the offspring the genesis of neurons that co-express CCR2 and melanin-concentrating hormone in the lateral hypothalamus (17), a neuropeptide implicated in excessive ethanol drinking (179). With current research showing low levels of ethanol exposure to increase drinking in offspring and produce changes in the immune system that ultimately affects neuronal function, future research on inflammatory systems could be very informative and important.

## Prenatal Nicotine Exposure

**Figure 5:**
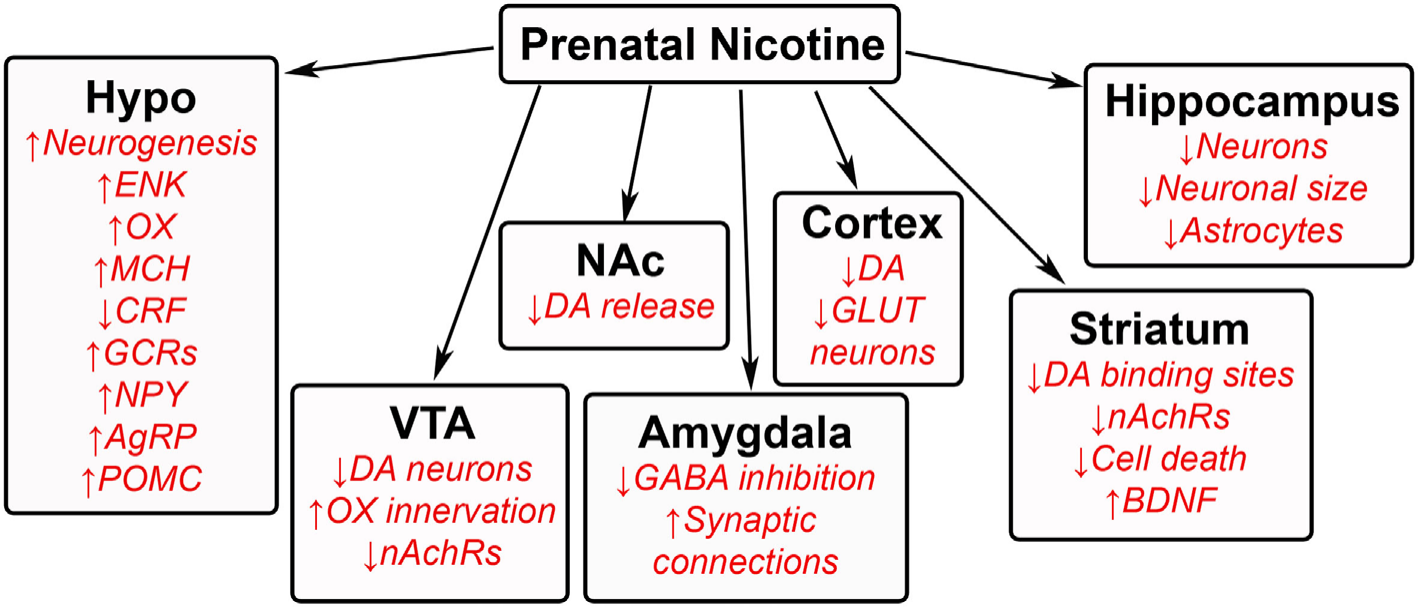
**Effects of prenatal nicotine exposure on offspring brain**. A schematic summarizing some of the changes that occur in the brains of offspring after being exposed to nicotine during gestation. GAL, galanin; ENK, enkephalin; OX, orexin; MCH, melanin-concentrating hormone; CRF, corticotrophin releasing factor; GCRs, glucocorticoid receptors; NPY, neuropeptide Y; AgRP, agouti-related protein; POMC, proopiomelanocortin; DA, dopamine; nAchRs, nicotinic acetylcholine receptors; GABA, γ-aminobutyric acid; GLUT, glutamate; BDNF, brain-derived neurotrophic factor.

The effects of prenatal nicotine exposure are broad in nature, affecting both behavioral and neuronal development in several regions of offspring brain. Human studies show that children exposed to tobacco during gestation exhibit an increased risk for tobacco use, craving, and withdrawal (180), as well as dependence (181). Animal studies similarly reveal increased nicotine self-administration and consumption in adolescent and adult offspring (182–185), along with increased ingestion of other substances including fat and ethanol (18). Additional behavioral problems include an increased risk of hyperactivity (186), impulsivity (185), and anxiety (34, 35). High levels of nicotine exposure are also associated with detrimental effects, such as growth retardation (187). While these nicotine studies lead one to question whether these changes are attributed to certain chemicals from the tobacco (188, 189) rather than to nicotine itself and result from social smoking as well as chronic smoking, the overall evidence clearly demonstrates that prenatal nicotine exposure negatively affects offspring. Similar to prenatal HFD and ethanol exposure, these changes in physiology and behavior induced by nicotine or smoking may be attributed to neuronal changes in the offspring brain (Figure 5).

### Prenatal Nicotine Exposure Alters Hypothalamic Neurocircuitry

Similar to dietary fat and ethanol, prenatal nicotine exposure has been found to affect the neuronal architecture and function of the hypothalamus. Several neuropeptides have been found to be altered in offspring during exposure to both low and high levels of nicotine. Some of the findings include a decrease in CRF and an increase in glucocorticoid receptors in the hypothalamus (190). They also show an increase in several orexigenic peptides, including neuropeptide Y, agouti-related peptide, and proopiomelanocortin in the arcuate nucleus (191), enkephalin in the medial hypothalamic paraventricular nucleus, and orexin and melanin-concentrating hormone in the perifornical lateral hypothalamus (18, 192). One of the more important findings from our lab shows that exposure to nicotine actually stimulates the genesis of neurons that express enkephalin, orexin, and melanin-concentrating hormone in the offspring hypothalamus (18), with these peptides positively related to the intake of nicotine (193). A small number of epigenetic studies also show changes in DNA methylation of the gene encoding brain-derived neurotrophic factor in human studies (194, 195), revealing the need for further epigenetic studies of specific cell types.

### Prenatal Nicotine Exposure Alters VTA–NA Center in Offspring

Prenatal nicotine exposure has been found to varying degrees to change neurons in the VTA and NA in offspring. With regards to the mesolimbic dopamine system, prenatal nicotine exposure decreases the number of dopaminergic neurons in the VTA (196), dopamine release from the NA (197, 198), and the number of dopamine binding sites in the striatum (199), altering the rewarding effects of nicotine in offspring. Neuronal connections to the VTA are also affected, with orexin innervation from the lateral hypothalamus to the VTA found to be increased (192). Additionally, prenatal nicotine reduces the number of nicotinic cholinergic receptor expression in both the VTA and NA core (196). Similar to the neurogenesis effect in the hypothalamus, prenatal nicotine increases cell survival in the NA and inhibits cell death related pathways (200), with this increase in cell survival consistent with the finding that prenatal nicotine exposure increases the nerve growth factor, BDNF (201). Further studies on this reward region in offspring will shed more light on how prenatal exposure reprograms offspring to become more prone to abusing nicotine.

### Prenatal Nicotine Has Global Effects on Other Areas of the Brain in Offspring

Prenatal nicotine exposure has been found to affect several other brain regions in offspring. In the hippocampus, this exposure decreases the number of neurons (202) while increasing the number of astrocytes (202), and it also decreases the neuronal area and cell size (203, 204), suggesting decreased hippocampal function. Similar effects are also found in the cortex of early postnatal rats (205), pre-weaned rats (204), and embryos (206), with studies revealing fewer glutamatergic neurons (207). These changes in the cortex induced by prenatal exposure have been linked to cognitive deficits and impaired executive control, causing rats to be more impulsive (208). Similar to the VTA and NA, dopamine levels are also decreased in the cortex of postnatal offspring (209). In the amygdala, one study found nicotine exposure to reduce the size of the amygdala in adolescent offspring (210), while a recent study from our lab has described an increase in neurogenesis and expression of enkephalin neurons in the central amygdala (18). With nicotine intake shown to generally reduce anxiety, future studies with prenatal exposure that relate behavior to amygdaloid function in offspring, as well as to other brain regions involved in decision making, would be interesting.

### Prenatal Nicotine Induces Epigenetic Changes in Offspring

Several studies show prenatal nicotine exposure to have epigenetic effects on peripheral organs in offspring. Prenatal nicotine has been found to decrease methylation on the promoter-expressing angiotensin receptor type 1a (211) and increase histone acetylation of the protein and fatty acid synthase in liver (212). Human studies have also reveal global changes in DNA methylation in offspring (213, 214). Evidence of a generational effect has also been shown in rat models, with maternal nicotine use and exposure during the prenatal period found to induce asthma and epigenetic changes in lungs of offspring that are two generations past the original exposure (215). This evidence suggests that the changes induced by prenatal nicotine exposure on brain neurochemical systems may be related to epigenetic changes occurring during development.

### Relationship between Nicotine and Inflammation

While reports of pure nicotine on adult systems generally reveal a reduction in inflammation (216, 217), several studies in humans show cigarette smoking to cause an increase in inflammation (218). Also, in a rat model, exposure to pure nicotine during the gestational period is found to increase the inflammatory mediators, IL-6 and TNF-alpha, in newborn blood serum (219). While this evidence is limited, it suggests the possibility that prenatal nicotine may have similar effects to HFD and ethanol exposure on inflammatory mediators, including CCL2.

## General Conclusion

The current knowledge of the neural control of ingestive behavior in offspring that are prenatally exposed to substances of abuse has come a long way from observational human studies. We are now only beginning to piece together how these changes in specific brain regions affect the overall neuronal communication within the brain. In addition, other systems of the central nervous system, such as glial cells, astrocytes, and oligodendrocytes, may also play a major role in this disturbed communication. More importantly is the emerging function of the immune system in the development of these neuronal systems in offspring and how substances of abuse disturb its actions. Future studies using these prenatal animal models will provide much insight in both the molecular and neuronal network changes as well as the mechanisms leading to these changes.

## Author Contributions

KP and SL prepared and revised the manuscript.

## Conflict of Interest Statement

The authors declare that the research was conducted in the absence of any commercial or financial relationships that could be construed as a potential conflict of interest.
